# A case of polycystic kidney disease infection caused by *Brucella*: report and literature review

**DOI:** 10.3389/fmed.2025.1613953

**Published:** 2025-07-10

**Authors:** Peng Su, Guotu Du, Ying Yang, Hong Zheng, Neng Zhang

**Affiliations:** ^1^Department of Urology, Affiliated Hospital of Zunyi Medical University, Zunyi, China; ^2^Department of Pathology, Affiliated Hospital of Zunyi Medical University, Zunyi, China

**Keywords:** *Brucella*, polycystic kidney disease, zoonosis, cyst infection, treatment

## Abstract

Polycystic kidney disease (PKD) is a common genetic disorder characterized by the progressive development of multiple renal cysts. While cyst infections in PKD are typically caused by common gut flora, infections due to atypical zoonotic pathogens like *Brucella* are exceedingly rare. We present a unique case of a 40-year-old male patient with PKD who developed a renal cyst infection caused by *Brucella* following exposure to livestock. Despite initial negative urine cultures, blood cultures confirmed the diagnosis of *Brucella* infection. The patient was successfully treated with a combination of doxycycline and rifampin. This case underscores the importance of considering zoonotic pathogens in patients with PKD and a history of livestock contact. It also highlights the critical role of early diagnosis and targeted antibiotic therapy in managing such rare infections. Additionally, this study provides a comprehensive review of brucellosis, covering its epidemiology, pathogenesis, clinical manifestations, diagnosis, and treatment strategies.

## Introduction

Polycystic kidney disease (PKD) is a prevalent genetic disorder, with a population incidence ranging from 1 in 2,500 to 1 in 1,000 individuals, affecting approximately 12 million people worldwide (1). The hallmark of PKD is the progressive development of multiple bilateral renal cysts of varying sizes. These cysts can compress renal tissue, ultimately leading to the destruction of renal architecture and the progression to end-stage renal disease (2). The disease is often asymptomatic in its early stages, making early detection challenging. However, as the disease advances, complications such as infection, hemorrhage, and obstruction may arise. Patients frequently experience recurrent urinary tract infections, which can be prolonged and involve the cystic lumen, leading to intracystic infections (3). The pathogens responsible for cyst infections are typically intestinal flora, such as *Escherichia coli*, with retrograde infection from the bladder through the ureter being the primary route (4). Nevertheless, non-intestinal flora pathogens can also cause renal cyst infections. Here, we describe a case of renal cyst infection caused by *Brucella*.

## Case presentation

The patient, a 40-year-old male, presented with a one-week history of right-sided flank pain, hematuria, and fever, with symptoms progressively worsening. On admission, physical examination revealed percussion tenderness in the right kidney area, but no tenderness was noted over the right ureteral region. Murphy’s sign was negative, and there was no migratory right lower quadrant pain or McBurney’s point tenderness. No palpable masses or lymphadenopathy were detected. The patient denied any history of tuberculosis, hepatitis, surgery, or trauma. He was initially diagnosed with polycystic kidney disease complicated by infection and was treated with antibiotics, hemostatic agents, and other symptomatic therapies.

**Figure 1:**
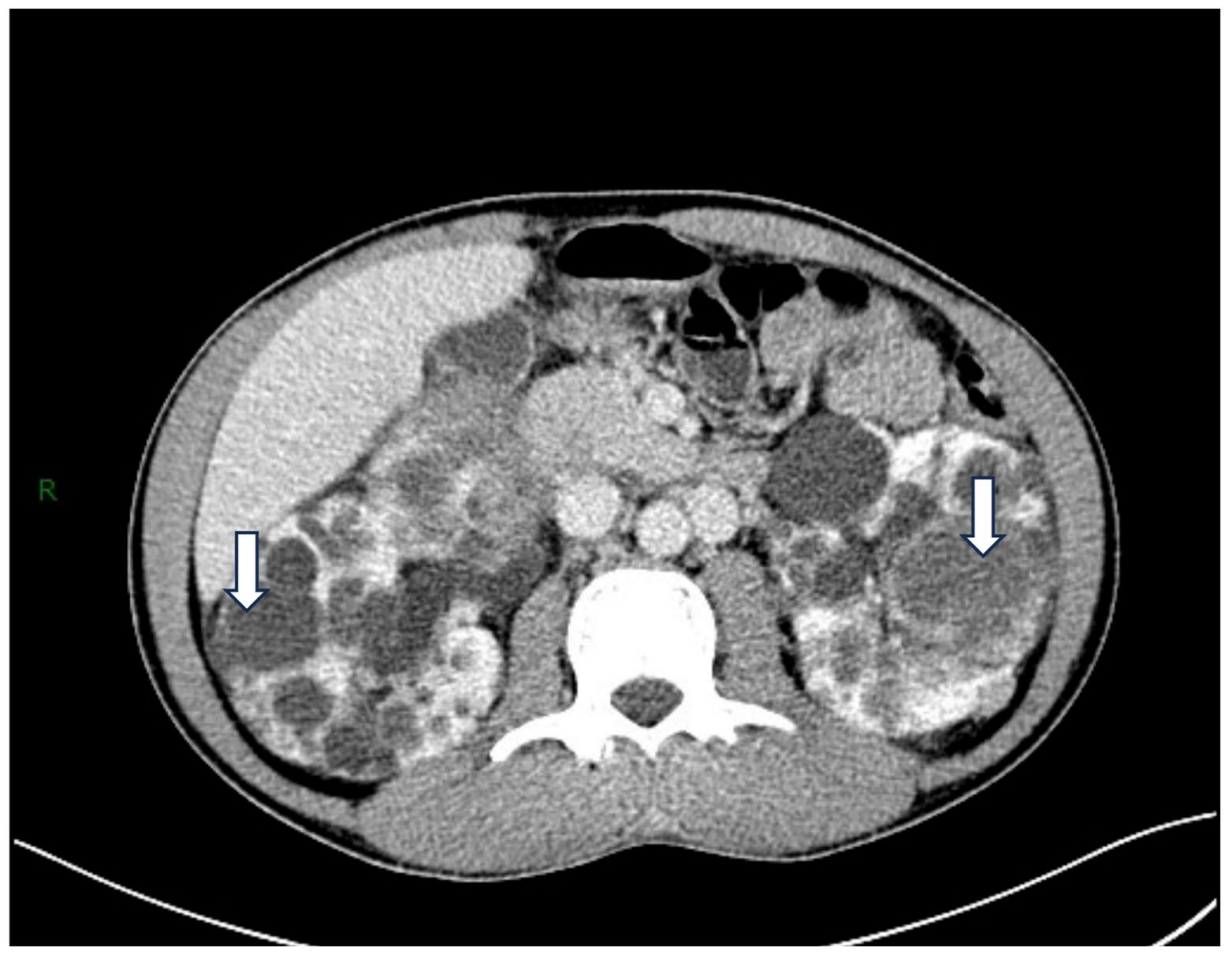
CT examination showed that the kidneys were enlarged in size and had irregular edges. Diffuse round-like cystic low-density shadows (white arrows) of varying sizes were seen in the kidneys. The renal cortex was thinned.

Upon admission, routine blood tests revealed a white blood cell count of 6.44 × 10^9/L, an absolute neutrophil count of 3.93 × 10^9/L (64%), an absolute monocyte count of 0.64 × 10^9/L (10%), and a hemoglobin level of 112 g/L. Urinalysis showed 25 leukocytes/μL, 116 red blood cells/μL, and a creatinine level of 118 μmol/L. The ultrasensitive C-reactive protein was elevated at 61.956 mg/L. A CT scan of the urinary tract revealed enlarged kidneys with multiple cystic lesions, consistent with polycystic kidney disease, with some cysts showing high-density areas or evidence of hemorrhage (Figure 1). The urine bacterial culture was negative.

Treatment: The patient had known allergies to cephalosporins and penicillins. Following urine and blood cultures, amikacin was administered for anti-infective therapy. During treatment, the patient developed a fever peaking at 39°C, with recurrent episodes that were not effectively controlled. Multiple urine cultures revealed no bacterial infection. On day 6 of treatment, blood cultures identified *Brucella* infection. The blood cultures were performed using an automated blood culture system, which triggered a positive alarm after 5 days of incubation. A sample from the mixed culture bottle was subcultured onto blood agar plates and incubated in a 5% CO₂ incubator. After 72 h, small, raised, round, grayish-white, and moist colonies with smooth edges were observed; Gram staining indicated Gram-negative coccobacilli. The urease test was positive, and the tube agglutination test with *Brucella* standard positive serum showed a titer of over 1:100, indicating the growth of *Brucella.* Further inquiry into the patient’s history revealed prior exposure to sheep farming. The treatment regimen was promptly adjusted to include doxycycline and rifampin. The patient’s fever gradually subsided, and upon discharge, he was instructed to continue oral doxycycline and rifampin to complete a six-week treatment course.

## Systematic review of brucellosis

### Overview of brucellosis

Brucellosis is a zoonotic infectious-allergic disease caused by bacteria of the *Brucella* genus.

*Brucella* is a Gram-negative, facultative intracellular bacterium renowned for its ability to evade the host immune system and adapt to a wide range of hosts. These bacteria can survive and replicate within the host’s mononuclear phagocyte system, interfering with immune responses through various mechanisms. In 1985, the World Health Organization classified *Brucella* into six species and 19 biovars based on host specificity and phenotypic characteristics: *Brucella melitensis (biovars 1, 2, and 3), Brucella abortus (biovars 1, 2, 3, 4, 5, 6, 7, and 9), Brucella suis (biovars 1, 2, 3, 4, and 5)*, and *Brucella* species associated with sheep, dogs, and the new tumor type (5). In recent years, three additional *Brucella* species have been identified: *Brucella pinnipedialis, Brucella ceti,* and *Brucella microti* (6, 7). The primary species pathogenic to humans are *B. suis, B. abortus, and B. melitensis* (8). Human infection typically occurs through direct contact with infected animal tissues (such as placentas, aborted fetuses), blood, urine, or milk, or through the consumption of unpasteurized dairy products or undercooked meat (9). Additionally, farm workers, slaughterhouse personnel, veterinarians, and laboratory staff are at risk of infection through inhalation of aerosols or mucosal contact (10). *Brucella* most commonly enters the human body through the skin or mucous membranes or the gastrointestinal tract, where it is phagocytosed by macrophages and proliferates within them. However, it usually does not directly infect renal cysts but rather enters them incidentally after hematogenous dissemination to the kidneys (3). Therefore, *Brucella*-induced renal cyst infection is clinically rare and easily overlooked or misdiagnosed. Although the mortality rate of brucellosis is low, the persistence of the pathogen can lead to chronic infection and complications, significantly impacting the patient’s quality of life. Thus, understanding the pathogenesis of *Brucella* and current treatment strategies is crucial for the effective prevention and control of brucellosis (Table 1).

**Table 1:** *Brucella* and its natural infection host.

Species	Prototype strain	Natural hosts	Other hosts	Zoonosis	Ref.
*B. meltensis*	16 M	Sheep, goats	Cattle, pig, horse	Yes	(46)
*B. abortus*	2,308	Cattle	Camels, sheep	Yes	(47)
*B. suis*	1,330	Pigs	Dogs, rodents	Yes	(48)
*B. ovis*	ATCC 25840	Sheep	Goats, cattle	No	(49)
*B. canis*	RM6/66	Dog	–	Yes	(50)
*B. neotomae*	5 K33	Desert wood rat	–	No	(51)
*B. pinnipedialis*	B2/94	Seals	–	No	(52)
*B. ceti*	TE10759-12	Porpoises, dolphins, whales	–	No	(53)
*B. microti*	CCM 4915	–	Rodents	No	(53)

### Epidemiology of brucellosis

Brucellosis is one of the most common zoonotic diseases globally. The incidence of brucellosis varies significantly across different regions. According to the World Health Organization (WHO), the global incidence ranges from 0.01 to 200 cases per 100,000 population (11). Recent studies have indicated that the global incidence is higher than previously estimated, with approximately 1.6 to 2.1 million new human cases reported annually (12). The Eastern Mediterranean region is one of the highest-incidence areas for brucellosis, with some areas reporting an incidence as high as 200.41 cases per 100,000 population. In contrast, the incidence in the Americas is relatively low. In Asia, the Middle East and Central Asia are high-incidence areas for brucellosis. For example, the incidence in Iran is 20.33 cases per 100,000 population, while in Kyrgyzstan, it is 12.45 cases per 100,000 population (11). Currently, the average annual incidence rate of brucellosis in China is 3.0 cases per 100,000 population. In recent years, the incidence of brucellosis has shown a complex trend globally. Effective public health measures have led to a decrease in the global incidence, from 26.83 cases per 100,000 population in 2015 to 1.83 cases per 100,000 population in 2020 (12). However, in regions with limited surveillance and intervention, the incidence remains high or has even increased. For instance, the incidence in Saudi Arabia rose from 10.19 cases per 100,000 population in 2015 to 14.17 cases per 100,000 population in 2017 (13).

### Pathogenesis of human brucellosis

**Figure 2:**
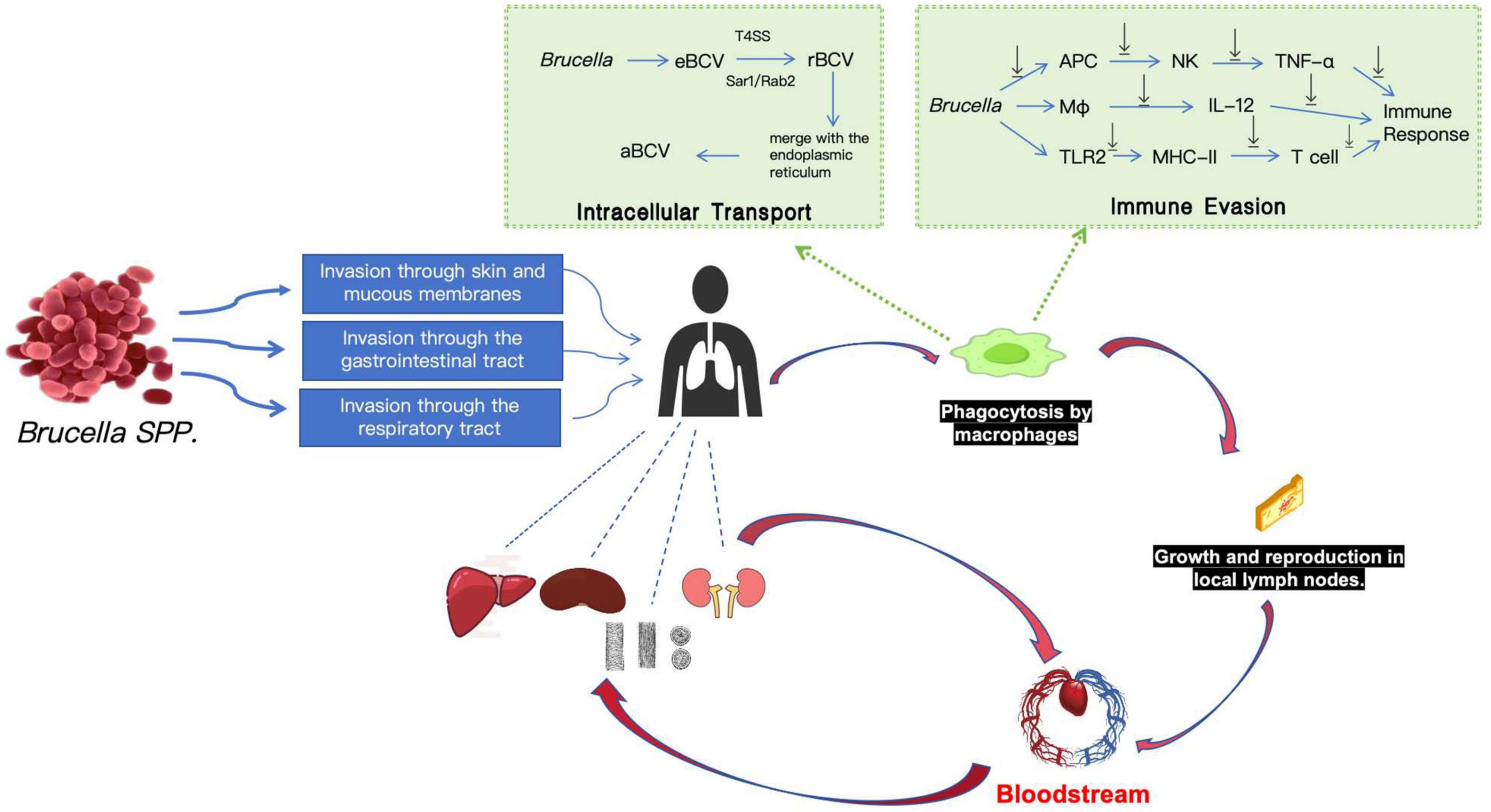
Pathogenesis of human brucellosis. *Brucella* enters the human body via the skin and mucous membranes, gastrointestinal tract, or respiratory tract. Upon entry, it interacts with lipid rafts on the host cell membrane and is subsequently engulfed by macrophages, forming an early *Brucella* -containing vacuole (eBCV). As the eBCV matures, some of these vacuoles evade lysosomal degradation and migrate to the endoplasmic reticulum (ER), where they fuse with the ER in a Sar1- and Rab2-dependent manner to form replicative *Brucella* -containing vacuoles (rBCVs). Within the rBCVs, *Brucella* proliferates extensively. In the later stages of infection, rBCVs, now densely populated with *Brucella*, transform into autophagic *Brucella* -containing vacuoles (aBCVs). These aBCVs release the pathogen into the extracellular environment through both lytic and non-lytic mechanisms, thereby completing the intracellular life cycle of *Brucella*. To evade immune clearance, *Brucella* employs multiple strategies. It inhibits the secretion of IL-2 by antigen-presenting cells, thereby preventing natural killer cells from producing pro-inflammatory cytokines such as IFN-*γ* and TNF-*α*. Additionally, *Brucella* disrupts IFN-γ -mediated phagocytosis, thereby evading immune attack. The pathogen can also interfere with the maturation of dendritic cells (DCs) by blocking the TLR2 signaling pathway and inhibit the secretion of IL-12 by macrophages, thus preventing DCs from activating T lymphocytes and impeding the establishment of a Th1 -type immune response. Initially, *Brucella* proliferates in local lymph nodes and establishes an infection focus. After approximately 2 to 3 weeks, the bacteria breach the lymph node barrier and enter the bloodstream, causing bacteremia. Subsequently, *Brucella* forms new infection foci in the reticuloendothelial system, including the liver, spleen, bone marrow, and lymph nodes, and may repeatedly re-enter the bloodstream, leading to recurrent episodes of the disease.

The pathogenicity of *Brucella* is closely linked to its ability to survive within host cells, with the cell wall playing a crucial role in this process. The *Brucella* cell wall consists primarily of an outer membrane and peptidoglycan. The outer membrane contains lipopolysaccharides (LPS), outer membrane proteins (such as lipoproteins and porins), and phospholipids (14). Compared to *E. coli*, *Brucella* LPS exhibits low endotoxin activity, strong resistance to macrophage degradation, and facilitates bacterial evasion of the host immune response. These properties enable *Brucella* to survive and proliferate within host cells, resulting in persistent infection (15). Figure 2 illustrates the pathogenesis of human brucellosis.

*Brucella* enters host cells by interacting with macrophage membranes via lipid rafts, forming a *Brucella*-containing vacuole (16). After entry, *Brucella* initially localizes to early phagosomes, where it avoids fusion with late endosomes and lysosomes, a crucial step in evading the host immune response. The acidic environment of the phagosome specifically induces the expression of the VirB operon, which regulates genes associated with the Type IV Secretion System (T4SS) (17). Through T4SS, *Brucella* delivers effector proteins to the host cell, altering the endosomal environment to promote bacterial survival and replication. Additionally, *Brucella* modifies its intracellular trafficking, transitioning from early phagosomes to autophagosomes and eventually reaching the endoplasmic reticulum (ER), where it fuses in a Sar1- and Rab2-dependent manner (18). Within the ER, *Brucella* replicates extensively without disrupting the host cell’s essential functions or inducing apoptosis. The ER supports *Brucella* replication within a single vacuole through continuous membrane production, leading to the formation of multiple *Brucella* replicons enclosed by the ER membrane, which are released through both lytic and non-lytic mechanisms (19). *Brucella* has specific survival mechanisms within macrophages and is protected by immune evasion mechanisms such as blocking macrophage apoptosis, inhibiting Th1-specific immune responses, and suppressing the production of tumor necrosis factor-*α* (TNF-α), making the pathogen difficult to eliminate.

Another mechanism by which *Brucella* survives within host cells is by altering the lipid composition of the phagosome membrane. *Brucella* expresses and secretes cyclic glucan synthase (CGS), which disrupts the lipid raft microdomains of the surrounding cell membrane, inhibiting phagosome maturation and preventing fusion with lysosomes, thus ensuring bacterial survival. This process occurs independently of the VirB operon (20). Additionally, oxidative killing is a key defense mechanism by which host phagocytes control intracellular pathogen replication, and *Brucella* has developed various strategies to counteract free radicals. It expresses two superoxide dismutases (SOD), SODA and SODC, with SODA involved in neutralizing free radicals and SODC protecting against superoxide anions produced during macrophage respiratory bursts (21). *Brucella* also expresses alkyl hydroperoxide reductase C to reduce peroxides, preventing oxidative killing, while its O-polysaccharide (O-PS) resists host cationic peptides and oxidative metabolites (22, 23). In summary, *Brucella* precisely regulates enzymes and secretory proteins at various stages to evade oxidative killing, allowing it to migrate to the endoplasmic reticulum and Golgi apparatus for replication.

### Clinical manifestations

The clinical manifestations of human brucellosis are diverse, most commonly presenting with fever, fatigue, headache, and muscle or joint pain. The severity and nature of symptoms vary depending on the *Brucella* species, the disease progression, and the affected organ systems. Infections caused by *Brucella melitensis* and *Brucella suis* tend to be more severe, while those due to *Brucella abortus* are generally milder. Due to the nonspecific symptoms, misdiagnosis is common, and if left untreated for more than 6 months, the disease can progress to chronic brucellosis, affecting multiple organ systems. The bones and joints are frequently involved, often presenting as sacroiliitis or spondylitis, though these symptoms are not specific. Digestive system involvement may manifest as hepatosplenomegaly (24). *Brucella melitensis* is particularly associated with orchiepididymitis, which may require surgery in severe cases (25). Gynecological conditions such as oophoritis and endometritis can also occur, potentially impacting fertility. Renal involvement may result in proteinuria. Neurobrucellosis, though rare, can present with neurological symptoms including meningitis and encephalitis (26). Cardiovascular involvement, while uncommon, may lead to serious complications and is the leading cause of morbidity and mortality in chronic brucellosis. Respiratory and endocrine involvement are less frequent but may present as pneumonia or thyroiditis. In conclusion, the clinical features of brucellosis are often nonspecific, complicating accurate diagnosis and treatment.

### Diagnosis of brucellosis

In recent years, the diagnosis of brucellosis has emerged as a topic of increasing interest and significance. The current diagnostic and main examination methods for brucellosis in WHO and China are shown in Tables 2, 3. The diagnosis of brucellosis primarily depends on the patient’s history of exposure in endemic areas, medical history, clinical presentation, routine laboratory tests, bacterial culture, and serological results. Laboratory tests often reveal normal or low white blood cell counts with relatively increased lymphocytes, though these findings lack diagnostic specificity. Bacterial culture remains the gold standard for diagnosing brucellosis, but it is time-consuming and may produce false negatives. Serological tests, which detect antibodies against *Brucella* lipopolysaccharides, face challenges such as cross-reactivity and the absence of standardized protocols. Molecular diagnostics, including PCR, are rapid and sensitive but similarly suffer from a lack of standardization. Immune cell markers, such as Toll-like receptors (TLRs), macrophages, T cells, and neutrophils, are involved in immune responses but have limited diagnostic utility (27–29). Protein markers like recombinant *Brucella* outer membrane protein 2b (Omp2b) demonstrate diagnostic potential (30). In terms of genetic markers, qPCR detection of specific *Brucella* gene fragments offers rapid, sensitive, and specific diagnosis. Additionally, microRNAs (miRNAs) and long non-coding RNAs (lncRNAs) are involved in the immune pathogenesis and may serve as diagnostic markers (31, 32). Advanced genetic testing methods, such as cgMLST and SNP analysis, improve the resolution of *Brucella* strain identification and hold promise for clinical diagnosis (33).

**Table 2:** Comparison of diagnostic methods for brucellosis.

Diagnostic method	Description	Advantages	Disadvantages	Ref.
Bacterial culture	Detection of live bacteria from blood or tissues	Gold standard, highly specific	Time-consuming, potential for false negatives	(54)
Serological tests	Detection of antibodies against *Brucella* antigens	Rapid, relatively easy to perform	Cross-reactivity, lack of standardization	(55)
PCR	Detection of *Brucella* DNA	Rapid, sensitive, specific	Requires specialized equipment, potential for contamination	(56)
Molecular diagnostics (qPCR)	Quantitative detection of *Brucella* DNA	Rapid, sensitive, specific	Requires specialized equipment, potential for contamination	(57)
Immune cell markers	Detection of immune cell responses	Reflects immune status	Limited diagnostic utility, non-specific	(27–29, 58)
Protein markers	Detection of specific bacterial proteins	Specific, potential for early diagnosis	Requires further validation	(30, 55)

**Table 3:** Comparison of brucellosis diagnosis and classification criteria between China CDC and WHO.

Diagnostic classification	China CDC	WHO	Ref.
Suspected case	Epidemiological exposure history + Clinical manifestations	Epidemiological exposure history + Clinical manifestations	(59)
Clinical diagnosis case	Suspected case + Preliminary diagnosis (e.g., Rose Bengal Plate Agglutination Test (RBT), Enzyme-Linked Immunosorbent Assay (ELISA), Gold Immunochromatographic Assay (GICA), Gram staining, etc.)	Suspected case + Preliminary diagnosis (RBT + Tube Agglutination Test (SAT) ≥ 160)	(59)
Confirmed case	Clinical diagnosis case + Confirmatory tests (e.g., SAT ≥ 100, Complement Fixation Test (CFT), Coombs test, bacterial culture, etc.)	Clinical diagnosis case + Confirmatory tests (e.g., ELISA IgG, Coombs IgG, bacterial culture, etc.)	(59)
Subclinical infection	Epidemiological exposure history + Confirmatory tests + Asymptomatic	No such classification	(60)

### Treatment strategies and current status of brucellosis

The current treatment regimen for human brucellosis remains based on the World Health Organization’s 1986 guidelines, which recommend doxycycline for 6 weeks combined with streptomycin for 2–3 weeks, or doxycycline with rifampin for 6 weeks (34). If streptomycin is unavailable or contraindicated due to allergic reactions, gentamicin may be used as an alternative (35). Studies indicate that combining doxycycline with streptomycin or rifampin reduces the minimum inhibitory concentration (MIC) of antibiotics within macrophages, achieving bacteriostatic levels. In cases complicated by arthritis, spondylitis, or endocarditis, a triple therapy consisting of streptomycin or gentamicin, doxycycline, and rifampin is typically prescribed for a minimum of 8 weeks, along with symptomatic treatment as needed (35). However, despite adherence to these regimens, the recurrence or clinical failure rate remains significant, ranging from 5 to 15% (34, 36).

The treatment of brucellosis mainly faces challenges such as antibiotic cell permeability, the bacterial replication niche within host cells, drug resistance, and the presence of antibiotic-resistant strains (37). To enhance antibiotic cell permeability, researchers have loaded antibiotics onto nanocarriers, adjusting surface charge to regulate circulation time and utilizing the high surface-area-to-volume ratio of nanocarriers for slow intracellular drug release, ultimately boosting antimicrobial efficacy (38–40). However, studies have shown that even increasing intracellular antibiotic concentrations does not completely suppress *Brucella* replication in macrophages. The bacteria remain viable and virulent after antibiotic treatment, posing a risk of re-infection (37). This persistence is likely linked to *Brucella*’s ability to establish a specialized replication niche within host cells, evading the phagolysosomal pathway and proliferating in a hostile microenvironment. Modifying this microenvironment, such as using hydroxychloroquine to raise the pH, has been shown to inhibit bacterial proliferation.

Bacterial drug resistance and the emergence of antibiotic-tolerant strains are additional factors contributing to treatment failure. Reports of antibiotic-resistant *Brucella* strains are increasing globally. In China, research on *Brucella* isolates has found that while most strains remain sensitive to commonly used therapeutic agents, some exhibit resistance to specific drugs. Prolonged use of antibiotics may also lead to the development of antibiotic-tolerant strains, which can survive repeated antibiotic exposure without acquiring a formal resistance mechanism. These strains may contribute to the chronic nature and recurrence of brucellosis.

### Prevention and control of brucellosis

Brucellosis not only causes substantial economic losses in the livestock industry but also poses a serious threat to human health. Despite prevention and control strategies proposed by the World Health Organization and the World Organization for Animal Health (OIE), brucellosis remains a major public health issue in many developing countries (41). Drawing from the experiences of developed regions like the European Union, brucellosis can be controlled and eradicated in stages at the source. Effective control measures include: (1) Enhanced monitoring and control: Monitoring helps reduce animal infections, identify infected groups, and prevent disease transmission through isolation (42). (2) Strict border quarantine: Controlling livestock movement and implementing rigorous border quarantine procedures, including testing imported animal products, prevents the introduction of infected animals (42). (3) Vaccination and test-slaughter strategy: Vaccination plays a crucial role in controlling brucellosis. Regular testing, combined with the immediate slaughter of infected animals, helps limit the spread of the disease (43). (4) Public education and legal measures: Raising awareness, particularly among farmers and herders, about proper protective practices through media campaigns, as well as implementing legal measures and financial compensation policies, is vital for effective disease control (44). (5) Laboratory quality management and biosafety: Ensuring proper management of laboratory personnel, equipment, sample handling, and testing procedures is essential, with attention to biosafety protocols. Given that *Brucella* species are among the pathogens that can be acquired in laboratory settings, strict adherence to biosafety level 3 (BSL-3) practices is necessary when handling *Brucella* cultures and samples. This includes the use of personal protective equipment (PPE), such as gloves, gowns, and eye protection, as well as the implementation of engineering controls like biological safety cabinets. Regular training of laboratory personnel on biosafety and biosecurity measures is also crucial to minimize the risk of laboratory-acquired infections. Global cooperation and localized implementation of these strategies are necessary for effectively controlling and ultimately eradicating brucellosis.

## Discussion

This case report describes a rare instance of renal cyst infection caused by *Brucella abortus* in a patient with polycystic kidney disease (PKD). The patient, a 40-year-old male, presented with typical symptoms of cyst infection, including right-sided flank pain and hematuria. Given the rarity of *Brucella* as an etiological agent in such cases, confirming it as the causative pathogen was of utmost significance. After the detection of *Brucella* via blood culture, the patient was promptly treated with doxycycline and rifampin, leading to a successful recovery. This case underscores the importance of considering atypical pathogens in complex renal cyst infections, particularly in patients with a history of livestock exposure or zoonotic infection risk.

Brucellosis manifests with a wide array of clinical symptoms, commonly including fever, fatigue, headache, and myalgia or arthralgia. These symptoms are often nonspecific, leading to potential misdiagnosis or delayed diagnosis. In this case, the patient’s chief complaints were right-sided flank pain and hematuria, symptoms akin to those of common urinary tract infections. However, the ultimate diagnosis of *Brucella abortus* infection through blood culture highlights the necessity in clinical practice to highly suspect brucellosis in patients with a history of livestock exposure and to conduct targeted testing promptly.

Brucellosis can be categorized into acute and chronic phases based on disease duration and clinical manifestations. The acute phase typically presents with fever, chills, sweating, and general malaise, whereas the chronic phase may be characterized by persistent or recurrent symptoms such as arthritis, spondylitis, or cardiovascular complications. In this case, the patient received treatment shortly after onset but experienced a prolonged period of symptoms due to diagnostic delay. This scenario suggests that for patients with persistent or recurrent symptoms, the possibility of chronic brucellosis should be considered, warranting a more comprehensive evaluation.

Although blood culture is the gold standard for diagnosing brucellosis, it is associated with limitations such as a lengthy turnaround time and the potential for false negatives. In this case, urine culture results were negative, while blood culture detected *Brucella* only on day 6. This diagnostic delay led to the ineffectiveness of the initial treatment regimen, with the patient experiencing persistent fever during treatment with amikacin. To enhance diagnostic reliability, it is recommended to employ a combination of serological tests (e.g., agglutination tests) and molecular biological methods (e.g., PCR) for a comprehensive diagnosis. Future research should focus on standardizing diagnostic protocols and extending follow-up periods to assess treatment efficacy and complications.

The treatment regimen for this case consisted of doxycycline and rifampin, which is one of the standard therapeutic regimens recommended by the World Health Organization. However, for more severe or recurrent cases, the addition of a third antibiotic, such as gentamicin or ciprofloxacin (45), may be necessary. In this case, the patient’s symptoms gradually subsided and eventual recovery was achieved after the treatment regimen was adjusted. This outcome demonstrates the critical importance of timely treatment regimen adjustment for improved prognosis. Moreover, in the event of drug-resistant strains, antimicrobial susceptibility testing should be conducted to guide individualized therapy.

The patient in this case had a history of exposure to sheep farming, which was crucial for the diagnosis. Brucellosis is primarily transmitted through contact with infected animals or consumption of unpasteurized dairy products. Therefore, in clinical practice, brucellosis should be highly suspected in patients with relevant exposure histories, and targeted testing should be conducted. Additionally, public health measures such as enhanced animal quarantine and vaccination promotion are of significant importance for controlling the spread of brucellosis.

## Conclusion

This case highlights the rarity and clinical significance of *Brucella* infection in patients with polycystic kidney disease (PKD). Accurate diagnosis was facilitated by meticulous medical history taking and prompt blood culture analysis, which led to the identification of the causative pathogen. The patient’s favorable response to targeted antibiotic therapy further validates the importance of precise diagnosis and appropriate treatment selection. This case underscores the necessity for heightened awareness of zoonotic pathogens in the context of renal cyst infections, especially in patients with a history of livestock exposure. It also reinforces the critical role of early diagnosis and adequate duration of antibiotic therapy in effectively managing such rare and complex infections. Collectively, these insights provide essential guidance for clinicians treating rare kidney infections in PKD patients, emphasizing the importance of considering atypical etiologies in the differential diagnosis.

## Data Availability

The original contributions presented in the study are included in the article/supplementary material, further inquiries can be directed to the corresponding authors.
